# NG2-proteoglycan-dependent contributions of oligodendrocyte progenitors and myeloid cells to myelin damage and repair

**DOI:** 10.1186/s12974-015-0385-6

**Published:** 2015-09-04

**Authors:** Karolina Kucharova, William B. Stallcup

**Affiliations:** Sanford Burnham Prebys Medical Discovery Institute, 10901 North Torrey Pines Road, La Jolla, CA 92037 USA

**Keywords:** Demyelination, Remyelination, Oligodendrocyte progenitors, Macrophages/microglia, Axon loss, NG2, CSPG4, Conditional knockout mice

## Abstract

**Background:**

The NG2 proteoglycan is expressed by several cell types in demyelinated lesions and has important effects on the biology of these cells. Here we determine the cell-type-specific roles of NG2 in the oligodendrocyte progenitor cell (OPC) and myeloid cell contributions to demyelination and remyelination.

**Methods:**

We have used Cre-Lox technology to dissect the cell-type-specific contributions of NG2 to myelin damage and repair. Demyelination is induced by microinjection of 1 % lysolecithin into the spinal cord white matter of control, OPC-specific NG2-null (OPC-NG2ko), and myeloid-specific NG2-null (My-NG2ko) mice. The status of OPCs, myeloid cells, axons, and myelin is assessed by light, immunofluorescence, confocal, and electron microscopy.

**Results:**

In OPC-NG2ko mice 1 week after lysolecithin injection, the OPC mitotic index is reduced by 40 %, resulting in 25 % fewer OPCs at 1 week and a 28 % decrease in mature oligodendrocytes at 6 weeks post-injury. The initial demyelinated lesion size is not affected in OPC-NG2ko mice, but lesion repair is delayed by reduced production of oligodendrocytes. In contrast, both the initial extent of demyelination and the kinetics of lesion repair are decreased in My-NG2ko mice. Surprisingly, the OPC mitotic index at 1 week post-injury is also reduced (by 48 %) in My-NG2ko mice, leading to a 35 % decrease in OPCs at 1 week and a subsequent 34 % reduction in mature oligodendrocytes at 6 weeks post-injury. Clearance of myelin debris is also reduced by 40 % in My-NG2ko mice. Deficits in myelination detected by immunostaining for myelin basic protein are confirmed by toluidine blue staining and by electron microscopy. In addition to reduced myelin repair, fewer axons are found in 6-week lesions in both OPC-NG2ko and My-NG2ko mice, emphasizing the importance of myelination for neuron survival.

**Conclusions:**

Reduced generation of OPCs and oligodendrocytes in OPC-NG2ko mice correlates with reduced myelin repair. Diminished demyelination in My-NG2ko mice may stem from a reduction (approximately 70 %) in myeloid cell recruitment to lesions. Reduced macrophage/microglia numbers may then result in decreased myelin repair via diminished clearance of myelin debris and reduced stimulatory effects on OPCs.

## Background

Damage to the myelin sheath not only impairs impulse transmission in central nervous system (CNS) axons but also leads to eventual loss of the neurons themselves [1, 2]. New oligodendrocytes for myelin repair are generated by so-called adult oligodendrocyte progenitor cells (OPCs) [3, 4] that represent the largest cycling population in the mature CNS [5–8]. Adult OPCs are effective in generating myelinating oligodendrocytes to repair lesions in experimental demyelination models and in the early stages of demyelinating pathologies. However, remyelination in chronic stages of multiple sclerosis (MS) is less impressive [9, 10], partly due to several factors in addition to OPCs and neurons that contribute to the disease process. For example, macrophages and microglia are key participants in the initial stages of myelin damage but are also required for the removal of myelin debris necessary to allow effective repair. Myeloid cells also produce factors that promote recruitment, proliferation, and differentiation of OPCs [11–13]. A more complete understanding of MS therefore requires delineation of the respective contributions of these various cell types to the disease process.

We have identified the NG2 proteoglycan, also known as chondroitin sulfate proteoglycan 4 (CSPG4), as a factor that influences developmental myelination and is also involved in both myelin damage and repair. During development, NG2 is required for effective expansion of the OPC pool. In germline NG2-null mice, reduced numbers of OPCs result in generation of reduced numbers of myelinating oligodendrocytes and to delayed myelination [14]. In a pathological model involving lysolecithin microinjection into the spinal cords of germline NG2-null mice, the extent of demyelination is reduced compared to demyelination seen in wild-type mice [15]. Nevertheless, myelin repair is also retarded in germline NG2-null mice. The complexity of these effects of NG2 ablation may be partly due to NG2 effects on three distinct populations of cells during demyelination and remyelination. NG2 is expressed by OPCs, macrophages/microglial cells, and microvascular pericytes associated with lysolecithin-induced lesions in the mouse spinal cord. Germline ablation of NG2 leads to reduced numbers of all three cell types in these lysolecithin-induced lesions [15], suggesting a role for NG2 in recruitment and/or expansion of these populations. In contrast, use of an experimental autoimmune encephalomyelitis (EAE) model has failed to detect NG2-dependent contributions of OPCs and myeloid cells to myelin damage and repair [16]. It is therefore important to more carefully analyze and dissect the roles of NG2 in the respective contributions of these cell populations to the demyelination/remyelination process.

In this report, we describe a Cre-Lox approach that generates OPC-specific NG2-null (OPC-NG2ko) and myeloid-specific NG2-null (My-NG2ko) mice. We show that, following lysolecithin-induced spinal cord demyelination, OPC-specific NG2 ablation negatively affects only the process of myelin repair, while myeloid-specific NG2 ablation reduces both myelin damage and repair. Our results begin to characterize the NG2-dependent changes in OPCs and myeloid cells that underlie the contrasting phenotypes of the OPC-NG2ko and My-NG2ko mice.

## Methods

### Animals

Animal work was performed in the AAALAC-accredited vivarium of the Sanford Burnham Prebys (SBP) Medical Discovery Institute according to guidelines approved by the Office of Laboratory Animal Welfare. All experimental protocols were approved by the SBP Institutional Animal Care and Use Committee. In order to generate cell-type-specific NG2-null animals, mice carrying loxP-flanked (“floxed”; fl) NG2 alleles were crossed with mice expressing Cre from the oligodendrocyte transcription factor 2 (Olig2) locus (Olig2^Cre^) or with lysozyme M-Cre (LysM^Cre^) mice. The experiments reported here utilize male control (NG2^fl/fl^), OPC-NG2ko (NG2^fl/fl^/Olig2^Cre^), and My-NG2ko (NG2^fl/fl^/LysM^Cre^) mice between the ages of 3 and 5 months. Mice with specific ablation of NG2 in OPCs were generated by crossing NG2 floxed mice [17, 18] with Olig2-Cre transgenic mice [19]. Olig2 is expressed in cells of the oligodendrocyte lineage, in motor neuron precursors, and in some radial glia. Since NG2 is expressed by OPCs, but not by motor neuron precursors or radial glia, the Olig2^Cre^ mouse is well-suited for ablating NG2 specifically in OPCs. Mice with myeloid-specific ablation of NG2 were generated by crossing NG2 floxed mice with LysM^Cre^ transgenic mice [20, 21], a well-established Cre driver line for gene ablation in myeloid cells.

### Lysolecithin-induced spinal cord demyelination

Spinal cord demyelination was carried out as previously described [15], using male mice (25–40 g) anesthetized with ketamine/xylazine (100/10 mg/kg) administered intraperitoneally. A 1.5-μl solution of 1 % lysophosphatidylcholine (L-α-lysolecithin; Sigma, St. Louis, MO) in 0.1 M phosphate buffered saline (PBS) was injected at a rate of 0.5 μl/min into the white matter between the Th12 and Th13 vertebrae, just lateral to the posterior spinal vein, at depths of 0.8 and 0.4 mm (1.5 min at each depth). The needle was left in place for an additional 2 min to avoid backflow. Sham-operated controls consisted of identical injections with 0.1 M PBS, followed by euthanasia at 7 days post-surgery. Some animals received intraperitoneal doses of 5-bromo-2-deoxyuridine (BrdU, 80 mg/kg) on post-surgery day 4, 3 days prior to euthanasia at day 7. For final analyses, animals were deeply anesthetized with ketamine/xylazine (100/10 mg/kg) and transcardially perfused with 0.1 M PBS, followed by 4 % paraformaldehyde (pH 7.4).

### Tissue preparation and immunocytochemistry

Spinal cords were removed after transcardial perfusion with 0.1 M phosphate buffer containing 4 % paraformaldehyde (pH 7.4). Tissue was post-fixed for 24 h at 4 °C and then cryoprotected for 24 h at 4 °C in 0.1 M phosphate buffer containing 20 % sucrose. Transverse sections (30 μm) were cut at −16 °C on a cryostat microtome (Cryocut, 1800) and collected free-floating in 0.1 M PBS containing 0.02 % sodium azide. For immunostaining*,* free-floating sections were first incubated for 60 min at room temperature in 0.1 M PBS containing 5 % normal donkey serum and 0.5 % Triton X-100. Sections were then incubated overnight at 4 °C with primary antibodies diluted in PBS containing 0.8 % Triton X-100, 0.02 % sodium azide, and 5 % normal donkey serum. In order to perform double or triple immunolabeling, respective primary and secondary antibody combinations were used sequentially. The following primary antibodies were used: (1) guinea pig or rabbit anti-NG2 (1:50 or 1:200) [22]; (2) rabbit or rat anti-platelet-derived growth factor receptor alpha (PDGFRα, [23] or eBioscience, 1:200); (3) mouse, rat, or rabbit anti-myelin basic protein (MBP, Sternberger MSMI 94, Invitrogene or Origene, 1:500); (4) rat anti-cluster of differentiation 18, integrin beta-2 (CD18; eBioscience, 1:200); (5) rabbit or goat anti-IBA1 (Wako, 1:1000, or Abcam, 1:500); (6) rat anti-F4/80 (Invitrogen, 1:100); (7) rat anti-BrdU (OBT0030G, Serotec, 1:50); (8) mouse anti-pan-axonal neurofilament (smi-312R, Sternberger, 1:1000); (9) rabbit anti-Olig2 (Abcam or PhosphoSolutions, 1:200); and (10) mouse anti-adenomatous polyposis coli (APC; clone CC1, Calbiochem, 1:50). After three 10-min washes with PBS, the sections were incubated with appropriate combinations of highly cross-adsorbed donkey secondary antibodies conjugated to Alexa488, CY3, and/or Alexa 647 (Jackson ImmunoResearch). Secondary antibodies were diluted 1:250 in the same solution as the primary antisera. For BrdU immunolabeling, sections were incubated in 2N HCl for 30 min at 37 °C, followed by boric acid neutralization (pH 8.5) for 10 min, and then processed via the immunostaining protocol described above. 4′-6-diamidino-2-phenylindole (DAPI, 4 μg/mL, D3571, Invitrogen) was used for general nuclear staining of all sections. After washing three times for 10 min with PBS, sections were mounted on slides, air-dried, and then cover-slipped with Vectashield (H-1000, Vector lab).

### Electron microscopy

Animals were transcardially perfused with 2.5 % glutaraldehyde plus 2 % paraformaldehyde in 0.1 M cacodylate (EM grade from Electron Microscopy Science) buffer (pH 7.4). Spinal cords were post-fixed with 1 % osmium tetroxide and embedded in Durcupan or Embed 812. Semi-thin (0.5 μm) and ultra-thin (60 nm) sections were prepared using Reichert-Jung ultramicrotomes. Toluidine-blue-stained semi-thin sections were examined by light microscopy (BX51 Olympus microscope equipped with an Optronics Microfire digital camera). Ultra-thin sections were examined by transmission electron microscopy using an FEI Technai Spirit G2 BioTWIN microscope equipped with a bottom mount Eagle 4k (16 megapixel) camera.

### Preparation of myelin

Crude myelin fractions from wild-type mouse brains were isolated by classical sucrose gradient centrifugation protocols [24]. In brief, brains were homogenized in 0.3 M sucrose and protease inhibitors. The homogenate was layered over 0.83 M sucrose and ultracentrifuged for 30 min at 75,000×*g* at 4 °C. Crude myelin was collected from the 0.3:0.8 M sucrose interface, resuspended in 20 mM Tris·Cl buffer (pH 7.45), and further purified by additional ultracentrifugation and two cycles of hypoosmotic shock.

### Bone marrow transplantation and preparation of bone-marrow-derived macrophages for phagocytosis assays

Wild-type and germline NG2-null mice on a β-actin-EGFP (enhanced green fluorescent protein) background were used as donors for bone marrow (BM) transplantation, as previously described [25, 26]. Gamma-irradiated wild-type and germline NG2-null mice served as recipients for EGFP-positive wild-type and germline NG2-null bone marrow, respectively. Bone marrow was harvested from euthanized wild-type and germline NG2-null β-actin-EGFP donor mice. Dissected femurs and tibiae were flushed with sterile 0.1 M PBS containing 5 mM EDTA and 2 % FCS, and red blood cells were lysed by an addition of two volumes of ACK buffer. Surviving BM cells were washed, filtered through a nylon mesh, and resuspended in sterile 0.1 M PBS containing 2 % mouse serum. Recipient mice each received 700,000 cells via retro-orbital injection. After a 6-week recovery, chimeric mice with at least 75 % engraftment were used for lysolecithin injection into the spinal cord. Infiltration of EGFP-positive cells into demyelinated lesions was assessed 1 week after lysolecithin injection.

For preparation of bone-marrow-derived macrophages, wild-type and germline NG2-null mice were euthanized by CO_2_ asphyxia, and BM cells were harvested as described above for BM transplantation. Following previously published protocols [25, 27], washed BM cells were adjusted to a density of 10^6^ cells/mL and cultured for 3 days in 10 % CO_2_ in a basic medium containing 1 nM IL-3 and 0.22 nM M-CSF (R&D and Peprotech, respectively). Non-adherent cells were collected, seeded at 6000 cells per well in 384 well tissue-culture plates (781901; Greiner Bio-one), and incubated in a basic medium with 0.44 nM M-CSF for four additional days. After washing with PBS, myeloid cells were incubated for 16 h in a serum-free macrophage medium (12065-074, Gibco). Phagocytic assays were performed on the following day by addition of 0.5 μg of crude myelin to each well. After 2, 4, 6, and 8 h of incubation, wells were washed, and cells were fixed with 4 % paraformaldehyde. Phagocytized myelin was quantified by double immunolabeling with MBP and CD18 antibodies.

### Image processing and quantification

At least four control, four OPC-NG2ko, and four My-NG2ko mice were examined for various aspects of demyelination and remyelination at each time point. Quantitative analysis of myelin and myelin debris in ventral and dorsal columns was performed in five transverse sections separated by intervals of 600 μm, thus spanning a distance of 2400 μm along the spinal cord (1200 μm on either side of the lesion). Comparison of MBP-positive volumes in the spinal cord did not reveal significant differences between non-operated and sham-operated animals, establishing that the sham operation did not affect the status of myelin. Sham-operated mice injected with 1.5 μL of 0.1 M PBS and surviving for 1 week were therefore used as controls for comparisons with all other lysolecithin-injected mice. Sections double-stained with antibodies against MBP and CD18 (or in some cases CD11b, F4/80, or ionized calcium binding adaptor molecule 1 (Iba1)) were scanned via confocal microscopy (LSM 710 NLO Zeiss; ZEN 2010), and colocalization of MBP-positive myelin debris in CD18-positive myeloid cells was evaluated using Image-Pro Plus 5.1 (Media Cybernetics). In order to quantify non-phagocytized MBP (i.e., intact myelin), phagocytized MBP pixels associated with CD18-positive cells were subtracted from total MBP pixels. Based on multiple observations, the intensity threshold representing positively labeled areas was chosen approximately 30 % above the background color. The average MBP-positive volume in sham-operated control Cre-negative mice was designated as 100 % and was used for comparison with MBP-positive volumes in all other experimental mice microinjected with either PBS or lysolecithin.

In order to compare axon number and the extent of axon myelination in control, OPC-NG2ko, and My-NG2ko mice 6 weeks after lysolecithin microinjection, three sections separated by intervals of 600 μm (thus spanning a distance of 1800 μm along the spinal cord) were double-labeled with pan-neurofilament (NF) and MBP antibodies. Axon abundance, axon diameter, and the extent of MBP-NF association were quantified by confocal microscopy in 1-μm optical sections using Image-Pro Plus software.

Identification of OPCs in lysolecithin-injected mice was complicated by our finding that PDGFRα was expressed not only by Olig2-positive OPCs but also by a population of Olig2-negative cells that appeared to arise from the meninges, as we have previously noted in spinal cord injury studies [23]. These Olig2-negative cells also continued to express NG2 in OPC-NG2ko mice, further distinguishing them from OPCs. While we suspect that these PDGFRα-positive non-OPCs may also contribute to the demyelination/remyelination phenotype, we did not wish to consider their impact in the current studies. We therefore eliminated these cells from our analyses by using double immunolabeling for PDGFRα and Olig2 to definitively identify OPCs.

OPC and myeloid cell proliferation was evaluated in sections immunostained for PDGFRα, Olig2, IBA1, and BrdU. Since PDGFRα and Olig2 are expressed on the cell surface and in nuclei, respectively, they were visualized using the same primary and secondary antibodies, while BrdU was visualized with a different primary-secondary antibody combination. In control, OPC-NG2ko, and My-NG2ko mice, mitotic indices for PDGFRα^+^/Olig2^+^ and IBA1^+^ cells were calculated by determining the percentage of cells in each of these two populations that were labeled for BrdU in single optical sections.

### Statistical analysis

Data were analyzed for statistical significance using un-paired *t*-tests and ANOVA. *P* values less than 0.05 were considered statistically significant.

## Results

### Cell-type-specific ablation of NG2

Previously, we found that germline ablation of NG2 affected the behavior of three different cell types during spinal cord demyelination and remyelination. Recruitment and abundance of oligodendrocyte progenitors (OPCs), pericytes, and macrophages/microglia were all diminished in germline NG2-null mice [15]. By using Cre-Lox-mediated ablation of NG2 in OPCs and in myeloid cells, our current study seeks to dissect the specific roles of NG2 in these two key cell types. For specific ablations of NG2 in OPCs (OPC-NG2ko) and in myeloid cells (My-NG2ko), NG2 floxed mice [17, 18] were crossed with Olig2^Cre^ [19] and LysM^Cre^ [20, 21] mice, respectively.

**Fig. 1:**
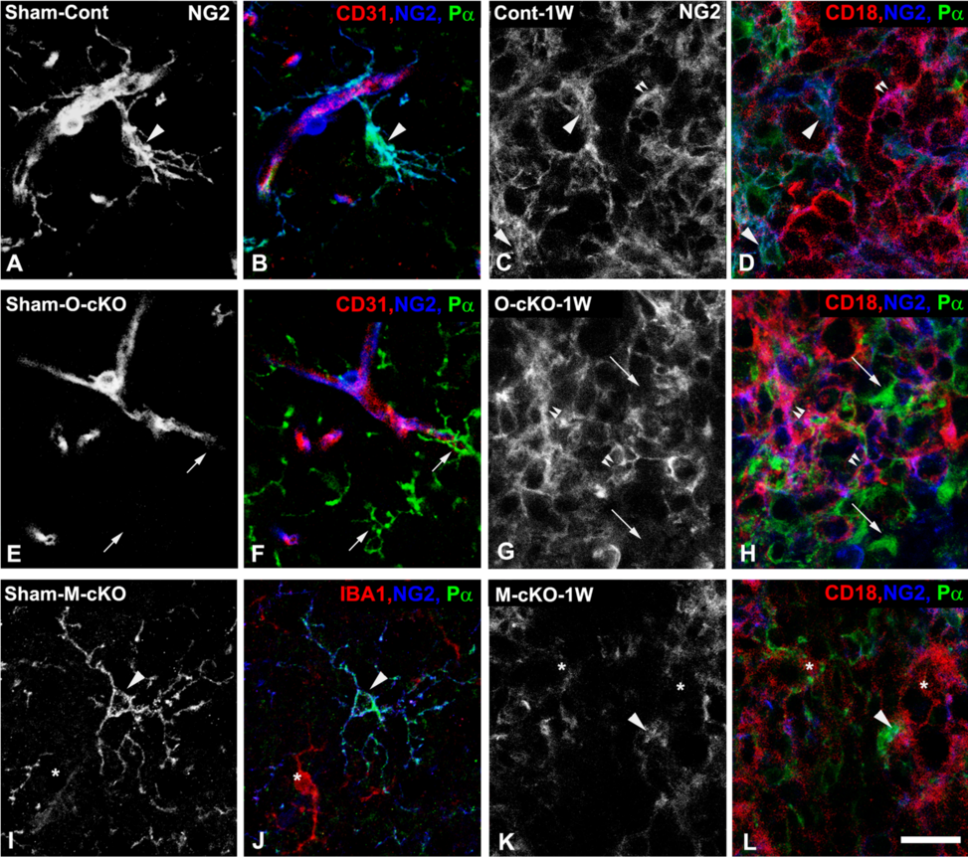
Cell-type-specific ablation of NG2. **a**, **b** *Sham-operated control mice*: **b** NG2 (*blue*) and PDGFRα (*Pα*, *green*) are both expressed by OPCs (*arrowhead*) in control mice injected with PBS (*Sham* *-Cont*). **a** To emphasize the presence or absence of NG2, NG2 alone is shown in *white* in the same field as **b**. **e, f** *Sham-operated OPC-NG2ko mice*: **f** NG2 (*blue*) is not detected in PDGFRα-positive OPCs (*arrows*, *green*) in OPC-specific NG2-null mice (*O-cKO*). **e** Same field as (**f**), NG2 alone (*white*). In both control and OPC-NG2ko genotypes, NG2 is expressed by pericytes observed in close proximity to CD31-positive endothelial cells (*red*; (**b**, **f**)). **i, j** *Sham-operated My-NG2ko mice*: **j** NG2 (*blue*) is expressed by *Pα*-positive OPCs (*green*, *arrowhead*) but is absent from IBA1-positive myeloid cells (*red*, *asterisk*) in myeloid-specific NG2-null mice (*M-cKO*). **i** Same field as (**j**), NG2 alone (*white*). **c**, **d** *Lysolecithin-injected control mice at 1 week post-injury:* **d** In demyelinated lesions in control mice 1 week after lysolecithin injection (*Cont-1W*), NG2 (*blue*) is expressed by *Pα*-positive OPCs (*green*, *arrowheads*) and by CD18-positive macrophages/microglial cells (*red*, *double arrowheads*). **c** Same field as (**f**), NG2 alone (*white*). **g**, **h** *Lysolecithin-injected OPC-NG2ko mice at 1 week post-injury.* **h** In demyelinated lesions in OPC-specific NG2-null mice 1 week after lysolecithin injection (*O-cKO-1W*), NG2 (*blue*) is ablated in *Pα*-positive OPCs (*green*, *arrows*) but is still expressed by CD18-positive myeloid cells (*red*, *double arrowheads*). **g** Same field as (**h**), NG2 alone (*white*). Compared to sham-operated animals, OPCs and myeloid cells are more abundant in demyelinated lesions. **k**, **l** *Lysolecithin-injected My-NG2ko mice at 1 week post-injury:* **l** In demyelinated lesions in myeloid-specific NG2-null mice 1 week after lysolecithin injection (*M* *-cKO-1W*), NG2 (*blue*) is expressed by *Pα*-positive OPCs (*green*, *arrowheads*) but is absent from CD18-positive myeloid cells (*red*, *asterisks*). **k** Same field as (**l**), NG2 alone (*white*). Scale bar is 20 μm

Our previous demonstration of OPC-specific NG2 ablation in the hypothalamus of OPC-NG2ko mice [17] is confirmed here by immunolabeling for NG2 and OPC-specific markers (PDGFRα and Olig2) in sham-operated and lysolecithin-injected spinal cords. Quantitative three-dimensional analysis of immunolabeled specimens from control (Fig. 1a–d), OPC-NG2ko (Fig. 1e–h), and My-NG2ko (Fig. 1i–l) mice indicates that the efficiency of NG2 ablation in PDGFRα^+^/Olig2^+^ cells in three OPC-NG2ko mice is 92, 95, and 98 %, respectively (average 95 %), whereas pericyte and myeloid cell expression of NG2 is not affected in these mice. In contrast, OPC expression of NG2 is unaffected in My-NG2ko mice. Myeloid-specific NG2 ablation in these mice, previously reported in the case of brain tumors [28], is confirmed here (Fig. 1) by comparisons of sham-operated and lysolecithin-injected spinal cords from control, OPC-NG2ko, and My-NG2ko mice via immunolabeling for NG2 and the myeloid markers CD18, CD11b, and F4/80, respectively. The analysis of NG2 ablation in myeloid cells is complicated by the fact that NG2 is not expressed by 100 % of any of these three myeloid populations in the lesions in control mice. In these controls, NG2 is found on 60 % of CD18-positive cells, 40 % of CD11b-positive cells, and 45 % of F4/80-positive cells. This may be due to the inherent heterogeneity of myeloid populations or because NG2 is expressed only transiently by myeloid cells (or both). Nevertheless, in lesions in My-NG2ko mice, NG2 expression is present on only 25 % of CD18-positive macrophages (Fig. 1l). Similar myeloid-specific absence of NG2 expression was noted using CD11b (18 % NG2-positive) or F4/80 (12 % NG2-positive) as myeloid cell markers (results not shown). A further complication is the likelihood of at least some phagocytosis of shed NG2 by myeloid cells. Since phagocytosed NG2 is difficult to distinguish clearly from cell surface NG2 on the basis of immunoreactivity, this might result in under-estimation of NG2 ablation in myeloid cells. In light of these uncertainties, the most conservative statement to be made is that NG2 is present on only 25 % of CD18-positive myeloid cells in My-NG2ko mice, compared to 60 % of CD18-positive cells in control mice. The significance of this reduction is supported by the resulting robust effect on myeloid cell recruitment in My-NG2ko mice.

### Kinetics of myelin damage and repair

**Fig. 2:**
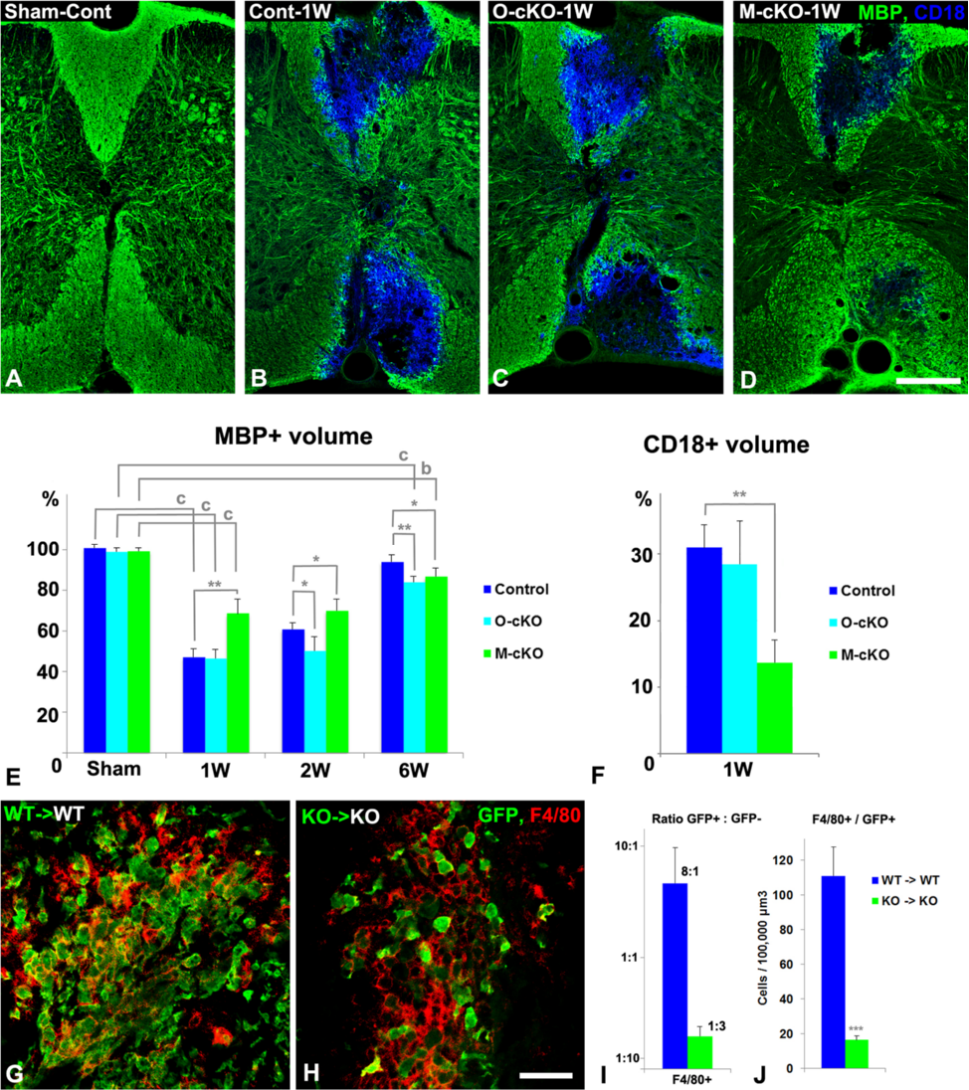
Quantification of myelin damage and repair and myeloid cell recruitment from bone marrow. One week after surgery, MBP (*green*) and CD18 (*blue*) expression are shown in control mice injected with PBS (**a**; *Sham-Cont*), and in control mice (**b**; *Cont-1W*), OPC-NG2ko mice (**c**; *O-cKO-1W*), and My-NG2ko mice injected with lysolecithin (**d**; *M-cKO-1W*). To quantify the extent of myelin damage, CD18-associated MBP (*cyan*) is subtracted from total MBP to provide a value for axon-associated myelin. **e** MBP-positive volumes are evaluated in control, OPC-NG2ko mice, and My-NG2ko mice 1, 2, and 6 weeks after lysolecithin microinjection, respectively. One hundred percent myelination is defined by the level of MBP labeling in sham-operated control mice. Demyelination is reduced by myeloid-specific, but not by OPC-specific, NG2 ablation. On the other hand, remyelination is retarded in both lines of NG2-null animals. **f** Significantly reduced infiltration by CD18-positive immune cells is seen in My-NG2ko mice compared to control or OPC-NG2ko mice. Wild-type (*WT*) mice transplanted with EGFP-positive wild-type bone marrow (**g**) and germline NG2-null (*KO*) mice transplanted with germline NG2-null bone marrow (**h**) were used for lysolecithin injections. Lesions were examined at 1 week post-surgery for co-localization of EGFP (*green*) and F4/80 (*red*). The recruitment of F4/80^+^EGFP^+^ cells to lesions in WT-to-WT chimeras is more than fivefold greater than that seen in KO-to-KO transplants (**j**). The 8:1 ratio of F4/80^+^EGFP^+^ to F4/80^+^EGFP^−^ cells in WT-to-WT chimeras falls to 1:3 in KO-to-KO chimeras as a result of reduced macrophage recruitment from the circulation (**i**). Quantification (**f**) represents the percentage of MBP-positive volume occupied by CD18 labeling. Values represent means ± S.D. Statistically significant differences evaluated by ANOVA and *t*-test are indicated by ^*^ *P* < 0.05; ^**^ *P* < 0.01; *** *P* < 0.001 when values were compared between controls and cell-type-specific (**e**, **f**) or germline (**j**) NG2-null mice at the same time point; ^*b*^ *P* < 0.01; ^*c*^ *P* < 0.001 indicate statistically significant differences within the same genotype at 1 and 6 weeks after lysolecithin injection. Scale bars represent 200 μm (**a–d**) and 50 μm (**g**, **h**)

Quantitative assessment of MBP-positive volumes in the spinal cords of sham-operated animals (Fig. 2e (Sham)) reveals very similar levels of overall myelination in control, OPC-NG2ko, and My-NG2ko mice. This is important for establishing that levels of myelination in the two lines of cell-type-specific NG2-null mice are not deficient prior to lysolecithin microinjection. One week after lysolecithin injection, the volume of the demyelinated lesion in OPC-NG2ko mice (Fig. 2c) does not differ significantly from the lesion volume seen in control mice (Fig. 2b). In contrast, the initial extent of myelin damage at 1 week in My-NG2ko mice (Fig. 2d) is significantly reduced compared to that seen in controls. These MBP volumes are quantified in Fig. 2e (1W). Figure 2e also shows that at 2 weeks post-injection, the initiation of lesion repair is evident in control mice (2W) and continues to improve at 6 weeks (6W). In contrast, lesion repair has not yet begun in OPC-NG2ko and My-NG2ko mice at 2 weeks and still lags behind repair in control mice at 6 weeks. In summary, myelin damage is reduced only in My-NG2ko mice, while myelin repair is diminished by cell-type-specific ablation of NG2 in both OPCs and myeloid cells.

### Macrophage recruitment and phagocytic function

Macrophage recruitment to lesions was evaluated 1 week after lysolecithin injection by immunolableling for CD18 (integrin subunit β2). The abundance of CD18-positive cells and the corresponding volume of infiltration in My-NG2ko mice (Fig. 2f) are greatly reduced compared to the values seen in control mice. In contrast, macrophage recruitment is not significantly altered in OPC-NG2ko mice. Similar results were obtained using CD11b (integrin subunit αM), F4/80, or Iba1 as macrophage markers, showing that the determination of myeloid cell abundance is independent of the choice of marker. Cell proliferation may also play a role in this distinction between control and My-NG2ko mice. As judged by BrdU incorporation, the mitotic index of IBA1-positive cells is reduced by 44 % in lesions in My-NG2ko mice, compared to control mice.

A similar reduction in macrophage recruitment to lesions is seen at 1 week post-injury in EGFP bone-marrow-transplanted mice. Compared to wild-type mice receiving EGFP-labeled bone marrow from wild-type donors, NG2-null mice receiving EGFP-labeled bone marrow from germline NG2-null donors exhibit a greatly reduced influx of EGFP-positive cells expressing the F4/80 macrophage marker (Fig. 2g–j). Moreover, the fact that F4/80-positive/EGFP-positive cells outnumber F4/80-positive/EGFP-negative cells by a ratio of 8:1 in WT-to-WT chimeric mice demonstrates that bone-marrow-derived macrophages, rather than resident microglia, comprise the majority of myeloid cells recruited to these lesions (Fig. 2g, i). By contrast, this macrophage:microglia ratio falls to 1:3 in KO-to-KO chimeric mice, reflecting the large decrease in macrophage recruitment to these lesions. These findings of reduced macrophage recruitment mirror the differences in demyelination observed via MBP labeling in Fig. 2e, suggesting a strong correlation between macrophage abundance and lesion severity. Data for macrophage recruitment are summarized in Table 1, row 3.

**Table 1:** Effects of cell-type-specific ablation of NG2 on cell and axon biology

Row		Control	OPC-NG2ko	My-NG2ko
	**Sham-operated animals**			
1	Number of Olig2^+^ cells	115.27 ± 3.8	111.02 ± 5.7	117.63 ± 5.4
2	Number of Olig2^+^/PDGF-Rα^+^ OPCs	14.55 ± 1.7	12.31 ± 2	14.46 ± 3.5
	**1 week after demyelination**			
3	Number of Iba1^+^ macrophages/microglia	117.19 ± 22.3	120.1 ± 28.1	39.64 ± 19.8**
4	Number of Olig2^+^/PDGF-Rα^+^ OPCs	42.92 ± 1.5	32.4 ± 1.7***	23.02 ± 7.2***
5	Mitotic index of Olig2^+^/PDGF-Rα^+^ OPCs (%)	22.91 ± 3.5	13.49 ± 1.5**	11.93 ± 2.1***
	**6 weeks after demyelination**			
6	Number of APC^+^ oligodendrocytes	62.14 ± 6.7	44.48 ± 8.6*	43.17 ± 4.1**
7	Well-myelinated axons	1056 ± 133	480 ± 128**	499 ± 80**
8	Myelin thickness (*g* value)	0.745 ± 0.089	0.861 ± 0.099***	0.87 ± 0.065***
9	Number of NF-positive axons			
	Small: diameter < 1 μm	1103 ± 60	660 ± 66***	812 ± 85**
	Medium: diameter = 1–2.5 μm	201 ± 29	135 ± 10*	118 ± 14*
	Large: diameter > 2.5 μm	18 ± 3	6 ± 1**	8 ± 2*

The key parameters for Olig2/PDGFRα-positive OPCs, Iba1-positive macrophages/microglia, and APC-positive oligodendrocytes were quantified in lesions in control, OPC-NG2ko, and My-NG2ko mice (*n* = 4 for each group) from 1 to 6 weeks after lysolecithin injection. The rationale for using Iba1 labeling in place of CD18 labeling was to attempt quantification of both activated myeloid cells inside lesions and non-activated myeloid cells remaining outside lesions. However, in this set of results, we have only examined Iba1-positive cells within lesions, in parallel to analyses of lesional CD18-positive cells in other parts of the text. Values represent means ± S.D. Statistically significant differences evaluated by ANOVA and *t*-test are indicated by ^*^ *P* < 0.05, ^**^ *P* < 0.01, and ^***^ *P* < 0.001 when values were compared between controls and NG2 conditional knockout mice at the same time point. Cell and axon numbers are determined in 100,000-μm^3^ volumes.

**Fig. 3:**
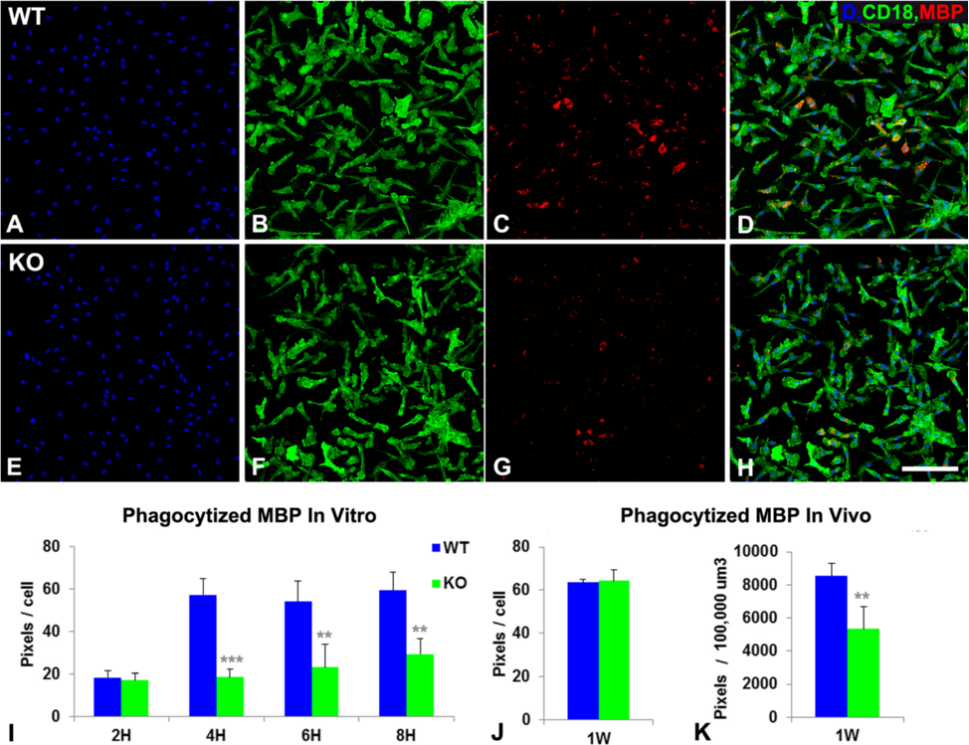
Myelin phagocytosis by CD18-expressing myeloid cells. Double immunolabeling for CD18 (*green*) and MBP (*red*) was used to quantify myelin phagocytosis by bone-marrow-derived macrophages in culture (**a–h**, DAPI in *blue*). In contrast to myelin phagocytosis by wild-type (*WT*) macrophages (**a–d**), NG2-null (*KO*) macrophages (**e–h**) exhibit reduced myelin phagocytosis after 4 h of incubation. Panel (**i**) quantifies these results over an 8-h time period (means ± S.D.). Panels (**j**) and (**k**) show a similar analysis of CD18/MBP double labeling in vivo at 1-week post-lysolecithin injection. Quantification is shown for both total MBP pixel density (**k**) and MBP pixels/CD18+ cell (**k**). **P < 0.01; ***P < 0.001. Scale bar indicates 100 μm for panels (**a–h**)

In addition to being responsible for the initial damage to myelin, a contrasting function of macrophages required for lesion repair is the phagocytic clearance of myelin debris. By double labeling for MBP and CD18, we were able to quantify phagocytized myelin within myeloid cells at 1 week post-injury (Fig. 3j, k). At this time point, overall phagocytosis of myelin is reduced by 40 % in My-NG2ko mice compared to controls (Fig. 3k). The fact that this difference is not seen when analyzed at the level of individual macrophages (Fig. 3j) could indicate that the overall difference in phagocytosis is based solely on reduced macrophage abundance in My-NG2ko mice. However, we have been able to detect a difference in phagocytic capability via in vitro comparisons of control and NG2-null bone-marrow-derived macrophages (Fig. 3a–i). Compared to controls, NG2-null macrophages appear deficient in their ability to phagocytize myelin when quantified on an MBP/cell basis. The fact that phagocytosis by control macrophages does not continue to increase after 4 h may indicate that macrophage capacity is saturated at this point, offering a possible explanation for the lack of a difference in MBP phagocytosis per cell in vivo; that is, after 1 week, NG2 macrophages may have had ample time to catch up with their wild-type counterparts.

### OPC abundance and proliferation in OPC-NG2ko and My-NG2ko mice

In the spinal cords of untreated adult OPC-NG2ko mice, there are small, statistically insignificant decreases in the number of OPCs and total number of Olig2-positive cells, compared to control mice (Table 1, rows 1 and 2). My-NG2ko mice are indistinguishable from control mice in this regard. Along with the indistinguishable MBP-positive volumes shown in Fig. 2e, these data are important for establishing that, prior to lysolecithin injection, all three mouse lines are very similar in terms of oligodendrocyte lineage cell populations and overall levels of myelination.

**Fig. 4:**
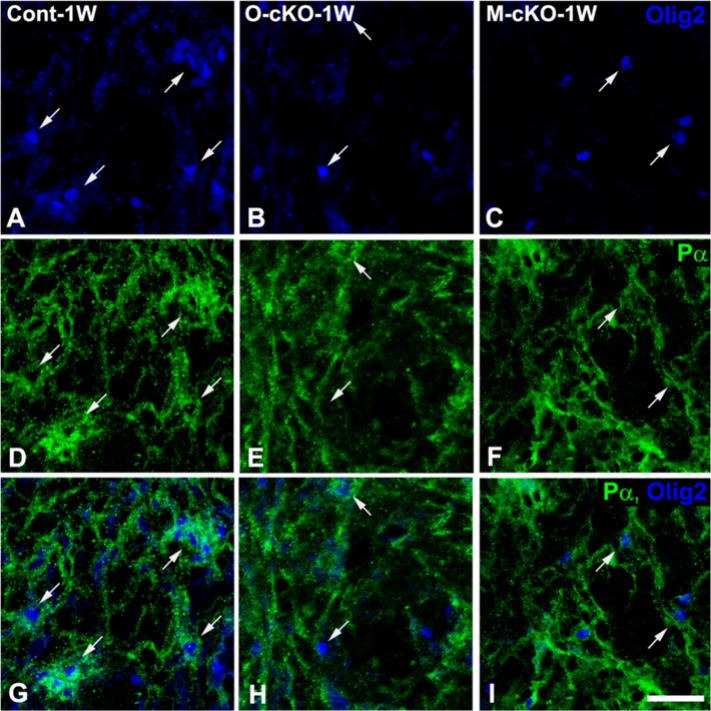
OPC abundance in lesions 1 week after lysolecithin microinjection. Larger numbers of PDGFRα (*Pα*, *green*)/Olig2-positive (*blue*) OPCs (*arrows*) are seen in lesions in control mice (**a**, **d**, **g**; *Cont-1W*) than in lesions in OPC-NG2ko (**b**, **e**, **h**; *O-cKO-1W*) or My-NG2ko mice (**c**, **f**, **i**; *M-cKO-1W*). Scale bar is 30 μm

**Fig. 5:**
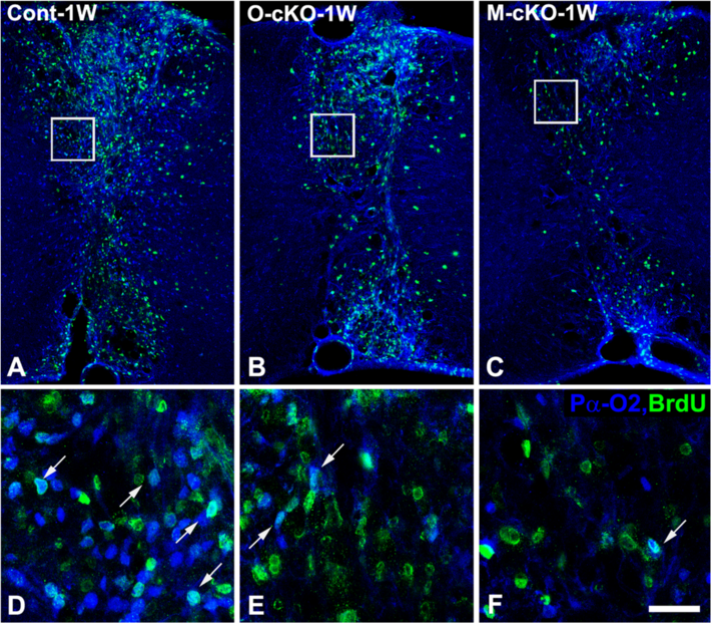
Proliferation of OPCs in lesions 1 week after lysolecithin microinjection. Larger numbers of proliferating BrdU-positive (*green*) PDGFRα-positive (*blue*, cell surface)/Olig2-positive (*cyan*, nucleus) OPCs (*arrows*) are seen in lesions in control mice (**a**, **d**; *Cont-1W*) than in lesions in OPC-NG2ko (**b**, **e**; *O-cKO-1W*) or My-NG2ko (**c**, **f**; *M-cKO-1W*) mice. The greatly reduced number of total BrdU-positive cells in M-cKO ( **c**) is due to significantly decreased numbers of macrophages/microglial cells. Scale bars are 160 μm (**a–c**) and 24 μm (**d–f**)

One week after lysolecithin injection, reduced OPC density is seen in lesions in OPC-NG2ko mice (Fig. 4b, e, h), compared to lesions in control mice (Fig. 4a, d, g). Somewhat surprisingly, OPC density is also reduced to a similar or even greater extent in lesions in My-NG2ko mice (Fig. 4c, f, i). These data are quantified in Table 1, row 4. In order to determine whether decreased OPC numbers are due to reduced OPC proliferation, we quantified BrdU incorporation into Pα^+^/Olig2^+^ cells at the 1-week time point. Interestingly, numbers of BrdU-positive OPCs are reduced in lesions in both OPC-NG2ko (Fig. 5b, e) and My-NG2ko (Fig. 5c, f) mice compared to lesions in control mice (Fig. 5a, d). Mitotic indices for OPCs are quantified in Table 1, row 5.

### Oligodendrocyte abundance and axon myelination in OPC-NG2ko and My-NG2ko mice

**Fig. 6:**
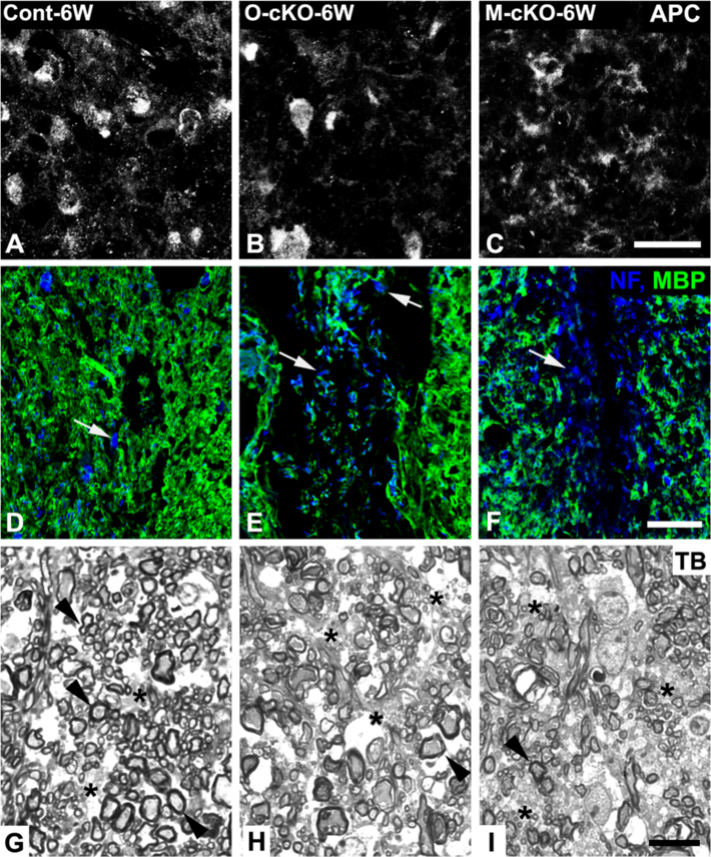
Regeneration of myelin. Six weeks after lysolecithin microinjection, fewer APC-positive cells are seen in lesions in OPC-NG2ko (**b**; *O-cKO-6W*) and My-NG2ko (**c**; *M-cKO-6W*) mice than in lesions in control (**a**; *Cont-6W*) animals. Also, reduced association of MBP-positive myelin (*green*) with pan-neurofilament-positive axons (NF, *blue, arrows*) is detected in the central region of lesions in OPC-NG2ko (**e**) and My-NG2ko (**f**) animals compared to lesions in control mice (**d**). In semi-thin sections, fewer well-myelinated fibers detected by toluidine blue staining (TB; *arrowheads*) are seen in lesions of OPC-NG2ko (**h**) and My-NG2ko (**i**) mice than in control (**g**) animals. Increased examples of poorly myelinated axons with thin, irregular myelin sheaths (*asterisks*) are detected in OPC-NG2ko and My-NG2ko mice relative to control animals. Scale bars indicate 20 μm (**a–c**), 30 μm (**d–f**), and 10 μm (**g–i**)

Reduced abundance of OPCs due to decreased proliferation might result in smaller numbers of mature oligodendrocytes available for myelin repair. We used immunolabeling for APC to assess numbers of mature oligodendrocytes at 6 weeks post-injury. Figure 6b, c reveal a reduction in oligodendrocyte density in both OPC-NG2ko and My-NG2ko mice, respectively, compared to controls (Fig. 6a). These results are quantified in Table 1, row 6.

**Fig. 7:**
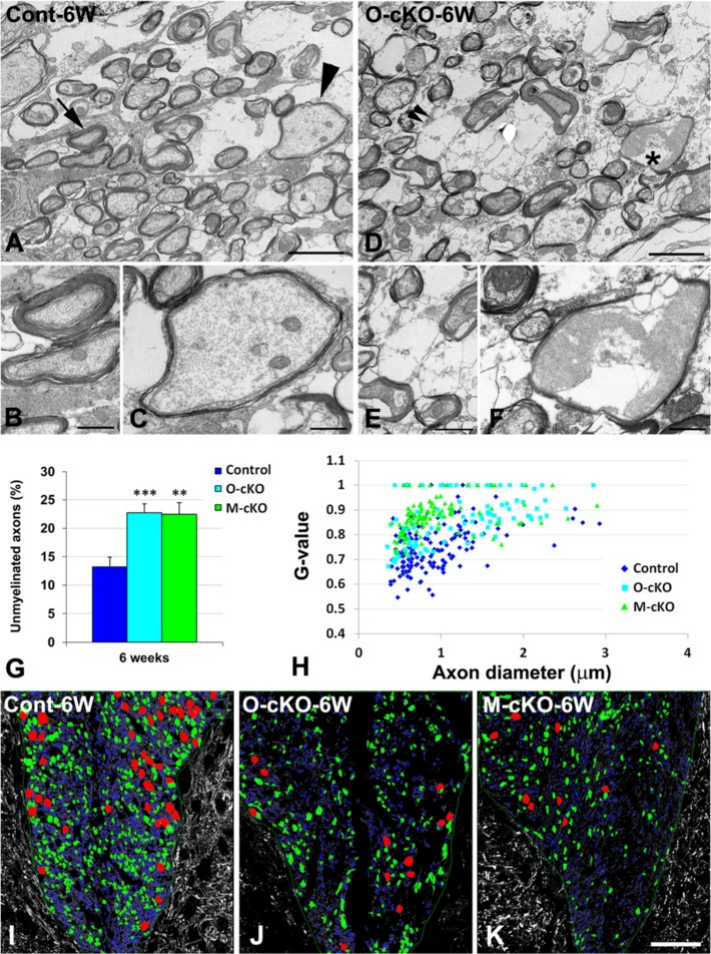
Remyelination and axon survival. Six weeks after lysolecithin microinjection, normally myelinated axons, characterized by pale axoplasm, neurofilaments, microtubules, mitochondria, and smooth endoplasm reticulum, are more numerous in lesions in control (**a–c**) than in OPC-NG2ko (**d–f**) mice. Panel (**a**) shows a well-myelinated axon (*arrow*, magnified in **b**) and a thinly remyelinated axon (*arrowhead*, magnified in **c**). Panel (**d**) shows an empty axon-like structure (*double arrowhead*, magnified in **e**) and an axon with degenerated axoplasm (*asterisk*, magnified in **f**). These structures are more numerous in OPC-NG2ko mice than in control mice. Quantitative analysis of pan-neurofilament-positive axons lacking association with MBP (from data represented in Fig. 6d–f) reveals increased numbers of unmyelinated axons in OPC-NG2ko and My-NG2ko mice (**g**). **h** Plotting *g* values (ratio of axon diameter to myelinated axon diameter) against axon diameter (**h**) reveals reduced myelin thickness (increased *g* value) in lesions in both OPC-NG2ko and My-NG2ko mice. In comparison with lesions in control mice (**i**), fewer NF-positive axons of all sizes are seen in lesions in OPC-NG2ko (**j**) or My-NG2ko (**k**) animals. *Blue*, *green*, and *red colors* indicate axons with diameters smaller than 1 μm, between 1 and 2.5 μm, and larger than 2.5 μm, respectively, as determined with image analysis software. Scale bars indicate 2 μm (**a, d**), 500 nm (**b**, **c**, **f**), 1 μm (**e**), and 40 μm (**i–k**)

A more detailed picture of axon remyelination at 6 weeks post-injury was obtained via double immunolabeling for MBP and NF, followed by quantification of the association between these two markers. In control mice (Fig. 6d), NF-positive axons are almost always seen in close association with MBP in the central area of the remyelinating lesion. However, in OPC-NG2ko (Fig. 6e) and My-NG2ko (Fig. 6f) mice, numerous NF-positive axons still lack association with MBP. These data are quantified in Fig. 7g. These findings are supported by examination of toluidine-blue-stained semi-thin sections. Compared to lesions in control mice (Fig. 6g), lesions in OPC-NG2ko (Fig. 6h) and My-NG2ko (Fig. 6i) mice contain 50 % fewer well-myelinated fibers (arrowheads) as well as increased numbers of axons with thin, irregular myelin sheaths (asterisks). These data are also presented in Table 1, row 7. Additional irregularities in lesional axons in OPC-NG2ko mice (Fig. 7d–f) are seen in electron microscopic comparisons with lesional axons in control mice (Fig. 7a–c). Determination of *g* values (axon diameter/myelinated fiber diameter) for lesional axons reveals an overall decrease in myelin thickness (increased *g* value) in OPC-NG2ko and My-NG2ko mice (Fig. 7h). The mean *g* value for control mice is 0.74, compared to 0.86 and 0.87 in OPC-NG2ko and My-NG2ko mice, respectively. (See also Table 1, row 8.)

An additional finding from the MBP/NF double labeling study is the reduced number of NF-positive axons present in 6-week lesions in OPC-NG2ko (Fig. 7j) and My-NG2ko (Fig. 7k) mice compared with control mice (Fig. 7i). The abundance of axons of all sizes is reduced by OPC-specific and myeloid-specific ablation of NG2. These data are quantified in Table 1, row 9.

## Discussion

Multiple sclerosis (MS) is a major cause of non-traumatic neurological disability among young adults in North America. During the acute phase of MS, damage to the blood-brain barrier allows infiltration of blood-derived cells that cause disruption of the myelin sheath surrounding axons in the central nervous system (CNS) [29–32]. This damage not only impairs axon ability to conduct impulses but also eventually leads to loss of the neurons themselves. Regeneration of myelin is not mediated by surviving oligodendrocytes but instead by new oligodendrocytes generated by oligodendrocyte progenitors [3, 4, 6]. This process works well in experimental models of myelin damage and repair but is often much less effective in complex demyelinating pathologies such as MS. Possible explanations for the incomplete remyelination seen in these complex pathologies include the failure of OPC recruitment into lesions, exhaustion of the proliferative capacity of OPCs, failure of demyelinated axons to interact with OPCs, and failure of OPCs to differentiate into mature oligodendrocytes [33–36]. These processes are also impacted by non-neural cell types that influence key interactions between OPCs and neurons. Improved treatment of MS requires a more complete understanding of factors that contribute to these various phenomena.

Our work with germline NG2-null mice has shown that genetic ablation of the NG2 proteoglycan negatively affects both developmental myelination in the early postnatal cerebellum [14] and remyelination of demyelinated lesions in the adult spinal cord [15]. Our work has further suggested that the involvement of NG2 in demyelination and remyelination may be more complex than its involvement in developmental myelination. This is due at least partly to the expression of NG2 by three distinct cell types that contribute in different ways to the properties of remyelinating lesions. NG2 expression is seen on OPCs, myeloid cells, and pericytes that are recruited to demyelinated lesions generated in the spinal cord white matter as a result of lysolecithin microinjection [15]. Use of Cre-Lox technology has allowed us to undertake dissection of NG2 roles in two of these cell populations: OPCs and myeloid cells. Cre-mediated ablation of NG2 in OPC-NG2ko and My-NG2ko mice has proved to be very efficient. At least 95 % of PDGFRα-positive/Olig2-positive OPCs are NG2-negative in OPC-NG2ko mice. NG2 ablation appears to be somewhat less effective in My-NG2ko mice, with 25 % of CD18-positive cells, 18 % of CD11b-positive cells, and 12 % of F4/80-positive cells remaining positive for NG2. This might be a real effect, due to lower efficacy of the LysM promoter compared to the Olig2 promoter [37]. However, it might also be partly artifactual due to myeloid phagocytosis of proteolytically shed NG2, contributing to association of NG2 immunofluorescence with CD18-positive cells. Regardless, there is a significant reduction of NG2 expression by myeloid cells in My-NG2ko mice, and the effect of NG2 loss on myeloid cell biology is quite robust.

Since we have previously reported that germline NG2 ablation causes transient, early postnatal deficits in developmental myelination [14], an important preliminary aspect of our studies has been establishing that the status of oligodendrocyte lineage cells and myelination in adult OPC-NG2ko and My-NG2ko mice does not differ significantly from that found in control mice. We suspected that this would be the case, based on the fact that developmental myelination in germline NG2-null mice appears to catch up with myelination in wild-type mice by the end of the third postnatal week [14]. However, for a more rigorous analysis, we demonstrate here that numbers of OPCs, total numbers Olig2-positive cells, and overall volume of myelination (judged by immunolabeling for MBP) are similar in control, OPC-NG2ko, and My-NG2ko adult mice. Questions regarding possible effects of the Olig2-Cre transgene on myelination have been previously answered by the finding that myelination is normal in Olig2/Olig2-Cre heterozygotes [38], which is the genotype used in our studies.

The extent of initial lysolecithin-induced myelin damage is not altered by OPC-specific NG2 ablation, indicating the lack of an NG2-dependent effect of OPCs on demyelination. This is not surprising in light of the absence of evidence in support of OPC involvement in demyelination.

In contrast to OPC-NG2ko mice, My-NG2ko mice exhibit deficits in myelin damage. At 1 week after lysolecithin injection, lesions in My-NG2ko spinal cords are roughly 40 % smaller than lesions in control and OPC-NG2ko spinal cords. The most likely cause for this reduced damage in My-NG2ko mice is the large decrease in recruitment of macrophages that contribute to demyelination. This decrease is also seen in bone marrow transplants from EGFP-positive wild-type and germline NG2-null donors, establishing that the effect on macrophage recruitment is independent of the mechanism of NG2 ablation. These bone marrow transplantation data also support the idea that the large majority of myeloid cells in lesions in wild-type mice is recruited from the circulation, since EGFP-positive myeloid cells outnumber EGFP-negative resident myeloid cells by an 8:1 ratio. A similar decrease in macrophage recruitment has been observed in our work with brain tumors in My-NG2ko mice [28], suggesting that impaired macrophage infiltration may be a general consequence of NG2 ablation in myeloid cells. The ability of NG2 to potentiate integrin activation [18, 39, 40] suggests the possibility that loss of NG2 might diminish the ability of macrophage integrins to bind to their endothelial ligands, with subsequent negative effects on macrophage extravasation into tissues. In support of this concept, we have used conformationally sensitive integrin antibodies to show that NG2 knockdown in macrophages diminishes the activation status of β1 and β2 integrins in the knockdown cells [41]. α4β1 and αMβ2 interactions with VCAM1 and ICAM1, respectively, on endothelial cells are central to leukocyte arrest and subsequent transmigration across the vascular endothelium [42–44]. It remains for us to demonstrate that NG2 knockdown also impairs macrophage transmigration of the vascular endothelium. In light of the importance of macrophage extravasation in normal immune surveillance and in multiple pathologies, understanding the involvement of NG2 in mechanisms that underlie transendothelial migration is an important goal.

Compared to control mice, myelin repair is slowed by the specific loss of NG2 from OPCs, suggesting an NG2-dependent loss of OPC function in OPC-NG2ko mice. The effect of NG2 ablation on OPC biology is initially apparent in the reduced mitotic index seen for OPCs in OPC-NG2ko mice. This decrease in OPC proliferation results in generation of a smaller than normal pool of progenitors at 1 week post-injury, leading to reduced numbers of mature oligodendrocytes at 6 weeks after injury. These findings in OPC-NG2ko mice are consistent with our reports of decreased OPC proliferation during both developmental cerebellar myelination [14] and spinal cord remyelination [15] in germline NG2-null mice. In addition, we have previously demonstrated the importance of NG2 for cell proliferation in several in vitro [18, 45–47] and in vivo [48–50] models. This suggests a general role for NG2 in promoting cell proliferation. This capability of NG2 is based on its ability to enhance both integrin and growth-factor-mediated signaling mechanisms that control cell proliferation [18, 45, 46, 51].

In spite of the reduced size of demyelinated lesions in My-NG2ko mice, myelin repair in these mice is also slow compared to control lesions. One explanation for this is suggested by the reduced OPC mitotic index observed in My-NG2ko mice. OPC proliferation is decreased in My-NG2ko mice to an even greater extent than in OPC-NG2ko mice, with correspondingly larger reductions in both mature oligodendrocytes and interactions of MBP-positive processes with axons. Reduced proliferation of OPCs in My-NG2ko mice could be due to the diminished numbers of macrophages, resulting in decreased production of cytokines/chemokines that affect various aspects of OPC biology [52–55]. However, the fact that My-NG2ko mice exhibit a larger deficit in OPC proliferation and abundance than seen in OPC-NG2ko mice indicates that an additional factor could also be at work. Since macrophages have an important role in phagocytosis of myelin debris [32, 56, 57], reduced macrophage infiltration in My-NG2ko mice might result in failure to clear myelin debris, leading to additional inhibition of OPC proliferation. In fact, our data demonstrate that overall phagocytosis of myelin debris is reduced by 40 % in My-NG2ko mice. Our in vivo results during the first week after the demyelination event do not detect impaired phagocytosis at the level of individual myeloid cells, suggesting that reduced clearance of myelin debris may be due solely to reduced macrophage recruitment in My-NG2ko mice. However, studies performed in vitro demonstrate that NG2-null macrophages are deficient in short-term phagocytosis of myelin compared to wild-type macrophages. These deficits in clearance of myelin debris might also contribute to diminished myelin repair, independent of changes in OPC proliferation. Additional work will be required to evaluate the mechanistic basis of NG2-dependent macrophage/microglia effects on OPC proliferation, clearance of myelin debris, and myelin repair. It is also possible that NG2 ablation has other effects on myeloid cell function. For example, in our previous study of lysolecithin-induced demyelination in germline NG2-null mice, we detected changes in cytokine levels indicative of a shift in macrophage polarization from a pro-inflammatory to anti-inflammatory phenotype [15]. It remains to be determined whether such changes in cytokine profiles can be demonstrated in My-NG2ko mice and whether these changes can be specifically linked to macrophages, as opposed to other cell types in the lesion.

Reduced remyelination in OPC-NG2ko and My-NG2ko mice is most apparent in specimens double-stained for NF and MBP to identify changes in interactions between axons and myelinating oligodendrocytes. At 6 weeks after the initial demyelination, many axons in the central area of lesions in OPC-NG2ko and My-NG2ko mice still lack association with MBP-positive oligodendrocytes, in contrast to the extensive MBP association with axons seen in control mice. These findings at the level of immunostaining are reinforced by higher power examination of lesions in semi-thin and ultra-thin sections from control, OPC-NG2ko, and My-NG2ko mice. Well-myelinated axons are less abundant in lesions in OPC-NG2ko and My-NG2ko mice. In addition, determination of *g* values for axons in these lesions reveals a statistically significant decrease in myelin thickness for axons in OPC-NG2ko and My-NG2ko mice.

It is extremely significant that our comparison of axon abundance in lesions in control, OPC-NG2ko, and My-NG2ko mice reveals the neuroprotective effect of OPCs and/or oligodendrocytes. At 6 weeks post-injury, the decrease in axon abundance across all axon size classes in the lesion center of OPC-NG2ko and My-NG2ko mice is well-correlated with the decreased number of oligodendrocytes, the diminished association of MBP with axons, and the reduced myelin thickness in these animals. This finding reinforces the concept that demyelination affects not only impulse conduction in axons but, more importantly, the survival of axons [1, 2, 58]. Prior to actual myelination, it is also possible that OPC association with axons has neuroprotective effects [59, 60].

In contrast to our studies with the lysolecithin-mediated demyelination model, previous use of an EAE model failed to detect a role for NG2 in demyelination and remyelination of the spinal cord [16]. Although this EAE study was able to document the participation of NG2-positive OPCs and leukocytes during myelin damage and repair, use of neither germline NG2-null mice nor wild-type mice transplanted with NG2-null bone marrow was able to demonstrate an effect of NG2 ablation at several levels, including disease severity and recovery, extent and repair of demyelination, and participation of OPCs and myeloid cells. Although it is not possible to provide a definitive answer that explains the different outcomes of the EAE and lysolecithin studies, we would point out key differences between the two animal models that may be contributing factors. Differences in the initiation, type, and chronicity of immune responses in the two models may underlie the differences in experimental results reported for the lysolecithin [15] and the EAE model [16]. EAE is an autoimmune-based model that engages both the adaptive and innate immune systems [61], producing fairly widespread damage to the CNS and resulting in a severe pathological phenotype. The lysolecithin model does not engage the adaptive immune system but relies on the action of the toxin to open the blood-brain barrier in a very focal manner, allowing engagement of the innate immune system [9]. The resulting lysolecithin-induced damage to myelin is very well-localized to the site of injection and results in very little pathological phenotype. An additional feature of the EAE model is the long-lasting nature of the innate immune response mediated by lymphocytes, resulting in frequent secondary attack and damage to areas under repair. While these phenomena are also characteristic of MS lesions, they inevitably complicate the detailed analysis of damage and repair in the EAE model. Thus, while the lysolecithin model could be considered to be at a disadvantage in terms of the absence of an adaptive immune component, it nevertheless has the distinct advantage of allowing exact localization of the site that needs to be analyzed, along with the ability to follow the somewhat simplified damage and repair processes. It is highly unlikely that our data would be as robust as they are if our analyses were made at a site that is peripheral to the primary site of the damage or if we could not identify whether lesions were in a damage or repair phase. Although we would not dispute the great utility of the EAE model in terms of its ability to mimic several key features of MS, the more random and recurring nature of damage in this autoimmune model makes it more difficult to choose optimal sites for detailed analysis. The extensive use of both types of models (and others) in published studies of myelin damage and repair illustrates the difficulty in choosing an optimal demyelination model but also supports the idea that each model has its respective strengths in terms of providing information relevant to myelin damage and repair.

In summary, our results demonstrate that NG2 is an important factor in OPC-dependent regeneration of white matter following spinal cord demyelination. Cell-type-specific ablations of NG2 reveal that the proteoglycan functions as both an OPC-intrinsic and OPC-extrinsic factor in promoting the OPC proliferation that precedes generation of mature oligodendrocytes and subsequent remyelination. In light of the key role played by macrophages/microglia in myelin damage and repair, the ability of NG2 to promote myeloid cell recruitment is an intriguing topic that merits further study.

## Conclusion

Using a lysolecithin microinjection model of spinal cord demyelination, we show that the NG2 proteoglycan is involved in the contributions of both OPCs and myeloid cells to the processes of myelin damage and repair. Specific ablation of NG2 in OPCs diminishes OPC proliferation, resulting in the generation of reduced numbers of mature oligodendrocytes and decreased remyelination of axons in lysolecithin-induced lesions. The specific ablation of NG2 in myeloid cells greatly reduces myeloid cell recruitment following lysolecithin-induced damage. Diminished macrophage numbers are responsible for a decrease in initial lesion size but also result in reduced lesion repair. This decreased repair may be due to reduced clearance of myelin debris in macrophage-deficient lesions but may also stem from the diminished OPC proliferation that is observed in the absence of macrophage-derived factors. Deficits in remyelination in both OPC-NG2ko and My-NG2ko mice are evident at two levels: (a) quantitative immunolabeling for MBP and (b) electron microscopic evaluation of myelin *g* values and numbers of myelinated axons. An important aspect of our results is the finding of reduced numbers of axons in incompletely remyelinated lesions in both OPC-NG2ko and My-NG2ko mice, emphasizing the importance of myelin for long-term axon survival.

## Abbreviations

APC: adenomatous polyposis coli
BrdU: bromodeoxyuridine
CD18: cluster of differentiation 18, integrin beta-2
CNS: central nervous system
DAPI: 4′-6-diamidino-2-phenylindole
EAE: experimental autoimmune encephalomyelitis
EGFP: enhanced green fluorescent protein
Iba1: ionized calcium binding adaptor molecule 1
ICAM1: intercellular adhesion molecule 1
L-α-lysolecithin: lysophosphatidylcholine
MBP: myelin basic protein
MS: multiple sclerosis
My-NG2ko: myeloid-specific NG2-null mice
NF: pan-neurofilament
NG2/CSPG4: neuron-glia antigen 2, also known as chondroitin sulfate proteoglycan 4
Olig2: oligodendrocyte transcription factor 2
OPC-NG2ko: OPC-specific NG2-null mice
OPCs: oligodendrocyte progenitor cells
PBS: phosphate buffered saline
PDGFRα: platelet-derived growth factor receptor alpha
VCAM1: vascular cell adhesion molecule
α4β1: integrin alpha-4/ beta-1
αMβ2: integrin alpha-M/ beta-2
